# Introduction. Statistical and computational challenges in molecular phylogenetics and evolution

**DOI:** 10.1098/rstb.2008.0182

**Published:** 2008-10-07

**Authors:** Nick Goldman, Ziheng Yang

**Affiliations:** 1 EMBL-European Bioinformatics InstituteWellcome Trust Genome Campus, Hinxton CB10 1SD, UK; 2 Department of Biology, University College LondonGower Street, London WC1E 6BT, UK

## 1. Introduction

Since the widespread acceptance of Darwin's theory of evolution (Darwin 1859), scientists have been interested in reconstructing the evolutionary relationships of living (and extinct) organisms. Evolution occurs too slowly to be experimented upon *in situ* in any but the most extreme cases, and scientists must look for information in the contemporary world that can lead to insights into the past. While perhaps the fossil record used to be the most obvious and explicit source of this information, starting from the early 1960s molecular sequences have taken over as the primary source of information on which to base reconstructions of the evolutionary history of life. The patterns of similarity and difference between the genomes of organisms related by descent from common ancestors implicitly hold vast amounts of information about species' relationships, and there is also considerable interest in describing and understanding the processes by which genomic sequences change over evolutionary time. The study of relationships of organisms and the study of the change of their genomes are intimately linked, and this has led to the forming of a coherent research community in molecular phylogenetics and evolution.

## 2. Times of plenty

**Figure 1:**
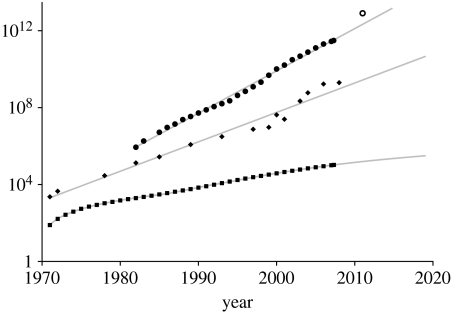
Growth curves relevant to research in molecular phylogenetics and evolution. The graph shows the growth over approximately 40 years of the total number of nucleotides stored in the EMBL Nucleotide Sequence Database (2008; circles), the number of transistors in current Intel PC processors (Intel 2008; Wikipedia 2008*b*; diamonds) and the cumulative citations found by searching for ‘molecular AND phylogen^*^’ in the ISI Web of Knowledge (WoK: http://www.isiknowledge.com/; searched on 24 June 2008; squares). Note the logarithmic scale on the *y*-axis. The sequence database and number of transistors per processor continue to grow exponentially, with doubling times of 16.4 months and 23.6 months, respectively. The open circle indicates the projection based on the additional sequencing effort as an outcome of the recently announced 1000 Genomes project (Spencer 2008). The citations for ‘molecular AND phylogen^*^’ in WoK are growing at an increasing rate, although slower than exponentially.

On 28–29 April 2008, the Royal Society hosted a Discussion Meeting entitled ‘Statistical and computational challenges in molecular phylogenetics and evolution’. Sixteen scientists from around the world presented their current research and visions for the future of the field, and this issue of the Philosophical Transactions of the Royal Society (Biological Sciences) records their and their co-authors' contributions. From the outset, molecular phylogenetic and evolutionary studies have relied on computers for the storing and analysis of sequence data, proteins and more recently DNA sequences and even entire genomes. Evolutionary analyses of molecular sequences pose many difficult mathematical, statistical and computational challenges (e.g. Felsenstein 1978; Yang *et al*. 1995), and the development of the research field has been closely linked to developments in computing technology. Most famously, Moore's Law (Wikipedia 2008*a*) predicts that computing power will double approximately every 2 years and figure 1 illustrates that this trend shows no sign of being broken after approximately 40 years. Less well known is the corresponding increase in the amount of data available to molecular phylogeneticists. The continuing trend for more and cheaper sequencing of genetic samples has generated an even more explosive increase: figure 1 shows that this too is exponential, with a rate even greater than Moore's Law. The size of the EMBL Nucleotide Sequence Database has been steadily doubling at approximately 16-month intervals since the early 1970s. The huge private and public effort to sequence the entire human genome forms a barely discernible bump in the growth curve around the year 2000 and the recently announced ‘1000 Genomes’ project, representing in a single project the sequencing of approximately six trillion DNA bases at a cost of $30–50 million (Spencer 2008), seems unlikely to look much more spectacular when compared with the projected underlying growth of this database.

While Moore's Law is impressive and the growth of sequence databases is more so, how has the field of molecular phylogenetics responded? The ISI Web of Knowledge (WoK: http://www.isiknowledge.com/) databases include Current Contents Connect (providing access to thousands of journals' bibliographic information), the Science Citation Index, ISI Proceedings (currently, records of nearly five million papers from over 60 000 conferences), biological abstracts from over 4000 life sciences journals, patent information and other scholarly sources. Figure 1 shows the growth of entries in WoK that are retrieved by searches using the term ‘molecular AND phylogen^*^’. We consider this a useful measure of ‘grass roots’ activity in molecular phylogenetics and evolution. While growth has been slower than exponential, it is still considerable. A similar curve is shown in this issue by Pagel & Meade (2008: figure 1), who concentrate on publications in scholarly journals; as expected, it takes some time for grass roots activity to be translated into peer-reviewed publications.

These are times of plenty for computational molecular phylogenetics and evolution—indeed, keeping up with the data deluge can be seen as one of the main challenges in the field. This is explicitly the *raison d'être* for the contribution of Rodrigo *et al*. (2008) to this issue. These authors give examples of challenging problems from their own research. Considering increased sequencing over time, they investigate the dynamics of sequence diversity, where modern studies can now look into the past of ‘sub-fossil’ remains (e.g. mammoths and ancient penguins, bison and chickens!). Increased sequencing over geographical location inspires investigation of ‘environmental shotgun sequencing’ whereby vast genomic samples from unknown organisms are collected in the type of global survey of life famously promoted by Craig Venter (Rusch *et al*. 2007). Finally, increased sequencing over species motivates a study of the ‘barcoding of life’, discussed in greater detail below.

Given that molecular phylogenetics is so dependent on computer technology, it has interested us for some years that specific research aims are sometimes achieved via increasingly complex analyses (using the growth of computer power), and sometimes by improved analytical, computational or approximate methods (to get more benefit within the constraints of existing computing power, or to cope with the growth of available data). In this issue, we see evidence of both approaches driving forward research into genome sequence evolution, as well as studies that attempt to quantify the effects of using simpler data analysis methodology where perhaps more complexity would be preferred.

## 3. Increased statistical and computational complexity

Increased complexity in the mathematical modelling of underlying biology is represented in a variety of subject areas. Cohen *et al*. (2008) devise probabilistic models of gene gain and loss in order to analyse genome-wide patterns of gene family presence and absence. They find that for individual gene families, rates of gain and loss are different, and that these rates also vary between gene families. Cohen *et al*. (2008) argue that the use of gene families increases the reliability of their data, filtering out confounding factors such as gene duplication, deletion of paralogs and horizontal gene transfer.

On a finer scale, Löytynoja & Goldman (2008) consider the ‘block-like’ structure of genomes: the fact that different regions (introns, exons, regulatory regions, etc.) exhibit different dynamics of evolutionary change. They exploit these differences to devise a multiple sequence aligner that can make allowance for the regional heterogeneity of evolutionary process to permit improved alignments, and can simultaneously estimate the regional structure of a genomic region in typical cases where this is unknown. Wang & Rannala (2008) also use fine-scale consideration of genome evolution to estimate recombination rates from population-level data. Whereas in the past it was only computationally possible to use methods based on approximations of the likelihood function, they now achieve considerable improvements to Bayesian Markov chain Monte Carlo algorithms when the exact likelihood is used.

Four further papers in this issue are concerned with adding biological reality to existing mathematical models of evolution in protein sequences or protein-coding DNA sequences. Improving these models has been a focus of molecular phylogenetics for many years, reflecting both that a successful model gives improved understanding of the processes of genome evolution and that a better description of evolutionary change is in turn expected to lead to more robust inferences of evolutionary relationships. Each of the four papers selects a different aspect of evolutionary biology for its inspiration. Choi *et al*. (2008) are concerned that existing models fail to match biological expectations. They construct evolutionary models that incorporate mutational bias and natural selection with predicted stationary distributions explicitly matching the distributions of sequences in databases. Huelsenbeck *et al*. (2008) examine different approaches to adapting standard models of amino acid replacement in phylogenetic analysis of protein sequences. These include Bayesian fitting of the general time-reversible model with approximately 200 parameters, as well as a mixture of commonly used empirical models.

Where Huelsenbeck *et al*. (2008) concentrate on developing models to accommodate the different evolutionary dynamics of different proteins, Pagel & Meade (2008) and Le *et al*. (2008) investigate how evolutionary models for DNA and proteins can deal with heterogeneity in the evolutionary process of specific sequences. Both consider mixture models. Pagel & Meade (2008) implement Bayesian mixture models to allow for the evolutionary rate to vary over time, while Le *et al*. (2008) develop mixture models in the likelihood framework to allow for heterogeneity in the dynamics of evolution among sites in the sequences.

## 4. Improved analytical, computational and approximate methods

Three papers in this issue aim to enable us to tackle larger problems in evolutionary sequence analysis by improving computational efficiency. Stamatakis & Ott (2008) present results on the efficient computation of the likelihood function on phylogenetic trees when we are analysing multiple genes, each of which is only known for some subset of all the species studied. This is the computationally expensive component in the most powerful tree estimation methods such as maximum-likelihood and Bayesian inference. The techniques they develop, implemented in their program RAxML, will be of particular value in phylogenomics, the phylogenetic analysis of datasets from many species and many genome regions.

Given an evolutionary tree, it is often interesting to map a specific trait onto the tree and thus to infer its evolutionary history. For example, methods for detecting the footprint of natural selection often consider the occurrences over evolutionary time of synonymous and non-synonymous point mutations in protein-coding genes, inferred from observed differences between contemporary sequences (e.g. Nielsen & Yang 1998)—we can place known sequences at the tips of a tree, and want to map synonymous and non-synonymous changes back in time. Minin & Suchard (2008) derive new analytical results for the mean number of state changes in a trait and the mean dwelling time in a given state. They give efficient algorithms to compute these properties, where previously time-consuming computer simulations were needed.

DNA barcoding is the assigning of DNA sequences of unknown origin to known species or taxonomic groups, and has applications in metagenomics, forensics, conservation genetics and molecular ecology. Full statistical approaches are too slow for current large-scale datasets (potentially hundreds of thousands of sequences). Munch *et al*. (2008) propose a novel heuristic approach based on the neighbour-joining method of tree reconstruction (Saitou & Nei 1987) and the non-parametric bootstrap (Felsenstein 1985). Such methods are particularly important as we enter the age of environmental shotgun sequencing discussed by Rodrigo *et al*. (2008).

## 5. Assessing the effects of oversimplicity

What are the consequences when we are forced, perhaps by lack of computing resources, to use data analyses that are less complex than we might prefer? Holder *et al*. (2008) and Whelan (2008) address this question in the context of estimating evolutionary relationships when the oversimplicity is in the mathematical model of sequence evolution. Both papers tackle this question using simulations of complex patterns of protein-coding DNA evolution, analysing the computer-generated data using a variety of current state-of-the-art methodologies that nevertheless are overly simple compared with the known ‘truth’ of their simulations. Such situations frequently occur, as we know existing inferential models of sequence evolution need improving (see §3 above) and computational power constraints also apply. Whelan (2008) concentrates on the effects of the genetic code, whereas Holder *et al*. (2008) focus on the effects of variations in the dynamics of sequence evolution from one site to the next. Both papers show that it is possible to make robust inferences under the conditions studied, typically by using relatively complex models closest to the simulation conditions. Without knowledge of the truth, however, there is no universal solution to the question of the best way to achieve optimal results in real data analysis, and the door is left open for further work.

Galtier & Daubin (2008) focus instead on the assumption that evolution of organisms follows a divergent tree-like structure. Different genes can lead to different inferences of evolutionary relationship; this may be due to analysis artefacts or may be biologically meaningful if, for example, the genes studied have been transferred other than by direct descent—a process known as horizontal gene transfer (HGT). Are species ‘trees’ still meaningful if there is significant HGT? Galtier & Daubin (2008) argue that they are, even for taxonomic groups such as the bacteria where HGT can be widespread and extensive.

## 6. And finally…

…we are delighted that this issue of the Philosophical Transactions of the Royal Society (Biological Sciences) contains two papers that develop the fundamental mathematics and statistics behind the computational analysis of molecular phylogenetics and evolution. Yang (2008) analyses a seeming paradox in Bayesian analysis of evolutionary trees, whereby with large amounts of data a full probabilistic inference of evolutionary relationships can become increasingly certain of an incorrect result. The problem arises when we try to choose between models (trees) that are nearly equally correct—or incorrect—and have very large amounts of data available, which is increasingly the case in molecular phylogenetics. It seems the problem is that with the increase of the data, the method's confidence increases faster than its accuracy, and Yang (2008) reports improved results when using newly developed prior distributions on the branch lengths of trees.

Concluding this issue, Klaere *et al*. (2008) introduce a new view of sequence evolution. The ‘one-step mutation’ (OSM) matrix describes how a single mutation in any branch of an evolutionary tree changes the character states observed at the tips of the tree. Sequence evolution, biologically comprising a succession of such mutations, is then modelled by multiplication of OSM matrices. This representation permits a linear algebra approach to the analysis of sequence evolution that is shown to unify maximum-parsimony, maximum-likelihood and distance matrix methods for phylogenetic inference, and leads to substitution mapping results closely related to those described by Minin & Suchard (2008).

The papers included in this issue provide a snapshot of theoretical developments in molecular phylogenetics and evolution. As the scientific world prepares to celebrate the 200th anniversary of Charles Darwin's birth and 150 years of his theory of evolution, they remind us that molecular phylogenetics and evolution remain a source of novel and challenging statistical and computational problems (Neyman 1971).
